# Evaluation of white blood cell count as a possible prognostic marker for oral cancer

**DOI:** 10.1186/1758-3284-3-13

**Published:** 2011-02-27

**Authors:** Astrid L Kruse, Heinz T Luebbers, Klaus W Grätz

**Affiliations:** 1 1University Hospital Zurich, Department of Craniomaxillofacial and Oral Surgery, Zurich/Switzerland

## Abstract

**Introduction:**

There seems to be increasing evidence that inflammation leads to cancer. For several cancers, an association with white blood cell (WBC) count has been reported. So far, no studies have been performed for cancer of the oral cavity and WBC. Therefore, the aim of the present study was to look at whether WBC count can be used as a prognostic marker for recurrence or metastases for oral cancer.

**Material and methods:**

For 278 patients with oral cancer, the preoperative WBC count was compared with the clinicopathological information: age, gender, T-status, N-status, recurrence, metastases, follow-up time, and time till recurrence or metastases appeared.

**Results:**

Out of 278 patients, 48 developed recurrence, 24 second tumors, 46 cervical metastases, and 14 distant metastases. The mean follow-up time was 35.97 months (range: 12-107 months). Significant Pearson correlation at the 0.05 level could be found for the T-status (0.046), but not for the N status (0.121). No significant correlation could be found between WBC count and the development of recurrence or metastases.

**Conclusion:**

In conclusion, our findings demonstrate that elevated WBC count does not seem to be a predictor for recurrence or for further metastases. Further research is recommended to investigate the WBC count in precancerous lesions and in HPV positive patients with oral SCC.

## Introduction

In 1863, Rudolf Virchow postulated the induction hypothesis that cancer originates at the site of chronic inflammation because he observed leukocytes in neoplastic tissues [1]. Associations between cancer and infections have been reported for several viruses, like human papilloma virus (HPV), human immunodeficiency virus (HIV), and chronic hepatitis B. Increasing evidence also suggests that inflammation may be linked to the pathogenesis of cancer like M. Crohn and colorectal carcinoma.

Some authors have observed an association between elevated serum C-reactive protein (CRP) levels and some cancers, like colorectal [2,3], lung [4] and head and neck [5,6]. Concerning WBC count as a predictor for cancer, several studies have been performed [7-9]. The stromal tissues of tumors have a high WBC count, and the inflammatory cell number and their cytokines production seem to correlate with tumor severity and prognosis [10].

For oral cancer, there is apparently no available data concerning WBC count. Therefore, the aim of the current study was to investigate the significance of preoperative WBC counts as a parameter for development of lymph node metastases or recurrence.

## Material and methods

Chosen for evaluation were 278 oral squamous cell carcinoma (SCC) patients (119 female, 159 male), with a mean age of 62.89 years, who were treated between 1999 and 2008 in the Department of Craniomaxillofacial and Oral Surgery, University Hospital Zurich. The WBC count came from a period of 1-5 days prior to surgical treatment. The following clinicopathological information was collected before data analysis: age, gender, T-status, N-status, recurrence, metastases, follow-up time, and time till recurrence or metastases appeared. The minimum follow-up time was 12 months. Exclusion criteria consisted of inadequate information and a follow-up time of less than 12 months.

Categorization of WBC was based on the distribution of WBC among the study participants: 2.5 - 4.79, 4.8 - 5.69, 5.7 - 6.79, 6.8 - 7.89, 7.9 - 9.99, 10.0 - 15.0 cells/μL. For statistical analysis SPPS^® ^18.0 (SPSS Inc, Chicago, IL) for the Mac^® ^was used, including the Pearson chi-squared test for the univariate analysis between the WBC count and the clinicopathological factors and crosstables.

## Results

**Figure 1:**
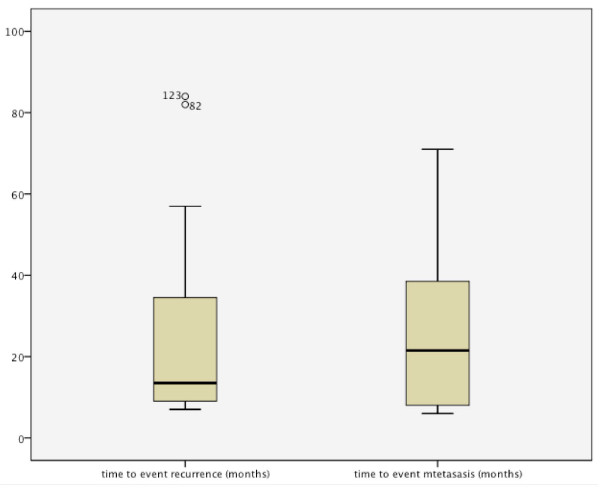
Comparison of time to recurrence and time to metastasis

Out of the 278 patients, 48 developed recurrence, 24 second tumors (Table 1), 46 cervical metastases, and 14 distant metastases (Table 2). The mean follow-up time was 35.97 months (range: 12-107 months); the mean time to event recurrence was 24.31 months (range: 7-84 months); and the mean time to event metastases was 18.27 months (range: 4-71 months) (Figure 1).

**Table 1:** Distribution of WBC count and recurrence

			recurrence			
				
			no recurrence	local recurrence	second tumour	Total
WBCcount	2.5-4.79	Count	19	5	4	28
		% within recurrence	9,2%	10,4%	16,7%	10,1%
	
	4.8-5.69	Count	25	6	2	33
		% within recurrence	12,1%	12,5%	8,3%	11,9%
	
	5.7-6.79	Count	47	8	6	61
		% within recurrence	22,8%	16,7%	25,0%	21,9%
	
	6.8-7.89	Count	43	9	3	55
		% within recurrence	20,9%	18,8%	12,5%	19,8%
	
	7.9-9.99	Count	45	13	7	65
		% within recurrence	21,8%	27,1%	29,2%	23,4%
	
	10.00-15.00	Count	27	7	2	36
		% within recurrence	13,1%	14,6%	8,3%	12,9%

Total		Count	206	48	24	278
		% within recurrence	100,0%	100,0%	100,0%	100,0%

**Table 2:** Distribution of WBC count and metastases

			metastases			
				
			no metastasis	cervical LN metastasis	distant metastasis	Total
WBCcount	2.5-4.79	Count	20	7	1	28
		% within metastases	9,2%	15,2%	7,1%	10,1%
	
	4.8-5.69	Count	22	9	2	33
		% within metastases	10,1%	19,6%	14,3%	11,9%
	
	5.7-6.79	Count	46	12	3	61
		% within metastases	21,1%	26,1%	21,4%	21,9%
	
	6.8-7.89	Count	47	4	4	55
		% within metastases	21,6%	8,7%	28,6%	19,8%
	
	7.9-9.99	Count	54	8	3	65
		% within metastases	24,8%	17,4%	21,4%	23,4%
	
	10.00-15.00	Count	29	6	1	36
		% within metastases	13,3%	13,0%	7,1%	12,9%

Total		Count	218	46	14	278
		% within metastases	100,0%	100,0%	100,0%	100,0%

**Figure 2:**
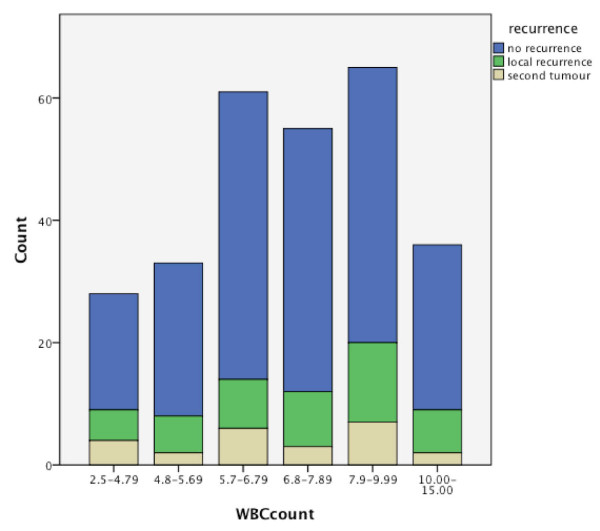
Distribution of WBC count and recurrence

**Figure 3:**
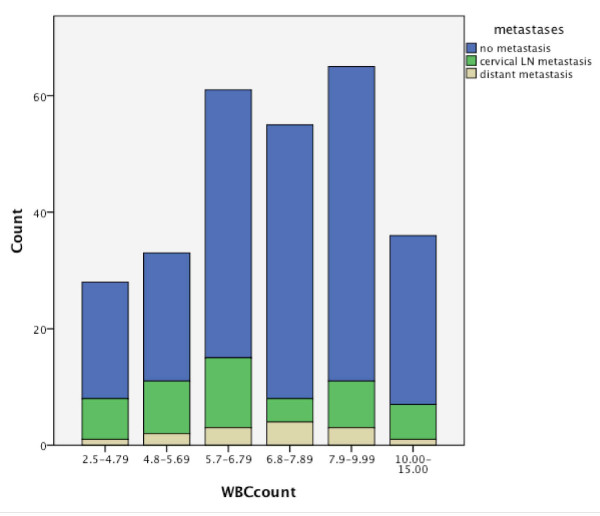
Distribution of WBC in regard to metastases

**Figure 4:**
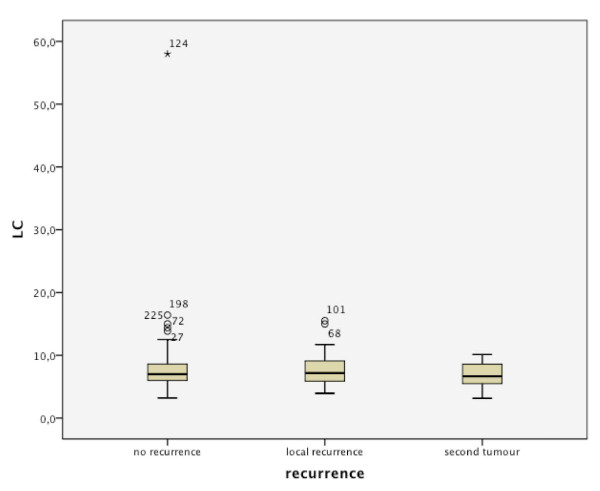
Comparison of recurrence/no recurrence in regard to WBC

**Figure 5:**
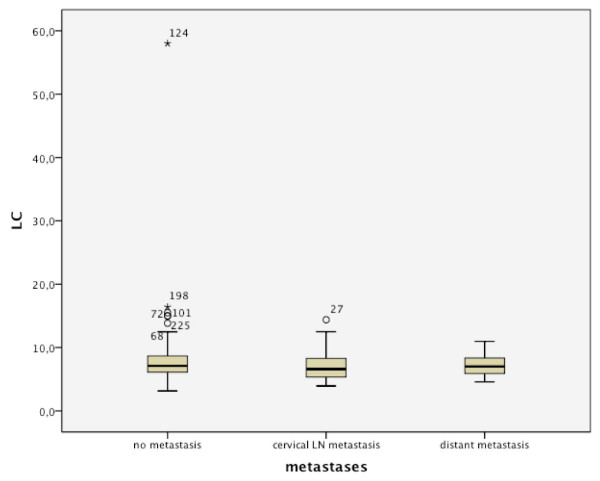
Comparison of metastasis/no metastasis in regard to WBC

The baseline distributions of WBC in regard to recurrence and metastases are shown in Figures 2 and 3. Out of 48 patients with local recurrence, 29 had an elevated WBC count of 6.8-15.0 cells/μL. From 46 patients with cervical metastases, 18 were in the elevated WBC count group, and 8 of 14 had distant metastases (Table 2). Significant Pearson correlation at the 0.05 level could be found for the T-status (0.046), but not for the N status (0.121). No significant correlation could be found between WBC count and the development of recurrence or metastases (Figures 4 and 5). This is also supported by a Pearson chi-squared test for univariate analysis, with an asymptomatic significance of 0.450 for recurrence and 0.459 for metastases.

## Discussion

In order to establish prognostic predictors for oral SCC, several studies have been performed. The purpose of our study was to attempt to discover a simple and cost-effective indicator for oral SCC. Based on the findings of this study, we concluded that WBC count is not a prognostic factor for recurrence of metastases. White blood cell count is highly variable because it is responsive to diverse acute and chronic stimuli. It is elevated by infection, by stress, and by chronic irritative exposures like smoking [11]. But due to its nonspecificity, WBC count can predict risk for multiple diseases besides cancer, coronary heart disease [12], or stroke [13].

For other cancers outside the head and neck, several studies have been performed. Grimm et al. (1985) [7] reported that the WBC count was significantly associated with risk of cancer death. And Erlinger et al. (2004) [14] were able to associate WBC count with total cancer mortality. Shankar et al. (2006) [9] also found an association between high WBC count and cancer mortality.

The evidence seems to be increasing that cellular proliferation in an environment rich in inflammatory cells, growth factors, and activated stroma is associated with the DNA damage that can potentiate the growth of cancer cells [1]. Non-steroidal, anti-inflammatory drugs may significantly reduce the risk of developing cancer, in particular that of the gastrointestinal tract [15]. Though no WBC count studies have been performed for oral cancer, several studies have been done for CRP: Gallo et al. (1995) [16]. demonstrated the significance of CRP and IL-6 with regard to tumor stage in 18 patients; Jablonska et al. (1997) [6] studied the CRP level, as well as IL-1β, IL-6, and TNF-α serum levels related to clinical stages of the disease in 42 patients; and recently Khandavilli et al. (2009) [5] found in a study of 60 patients that the CRP level is associated with worse overall outcome. The only correlation that could be found in the present analysis was for T status and WBC, and that seems to have no clinical relevance.

In 2009, Ki et al. [17] reported a significant correlation between the presence of acute mucositis and the CRP level in 40 patients during radiotherapy for primary laryngo-pharyngeal cancer. Therefore, it would be of interest--not only in mucositis patients, but also in precancerous lesions that are associated with inflammation, like erosive lichen--to investigate the relation between inflammatory markers like CRP and WBC counts.

Several limitations to this analysis must be considered. First, a WBC count analysis was performed only once; multiple measurements would have increased the precision of the results. Although WBC count is variable from day to day, a single measurement has been shown to predict risk of death for specific diseases, including cancer and cardiovascular disease [18]. Second, data on potentially confounding factors, such as medicinal use of aspirin or other NSAID, were unavailable.

Despite the potential limitations, the current study has several important strengths. First, this is the first study, to the authors' knowledge, dealing with the association of WBC count and oral cancer. Second, a relatively long follow-up time with a mean of 35.97 months was analyzed. And third, special attention was paid to second tumors and distant metastasis in addition to local recurrence and local metastases.

## Conclusion

In conclusion, our findings demonstrate that elevated WBC count does not seem to be a predictor for recurrence or further metastases. Further study is needed to investigate the WBC count in precancerous lesions and in HPV positive patients with oral SCC.

## Competing interests

The authors declare that they have no competing interests.

## Authors' contributions

ALK carried out the restrospective study and drafted the manuscript, HTL participated in the design of the study, KWG participated in the design and coordination of the study. All authors read and approved the final manuscript.
